# Novel Application of Behavioral Assays Allows Dissociation of Joint Pathology from Systemic Extra-Articular Alterations Induced by Inflammatory Arthritis

**DOI:** 10.23937/2469-5726/1510033

**Published:** 2016-06-30

**Authors:** Ann K Harvey, Mariah J Lelos, Claire J Greenhill, Ashley T Jones, Susanne P Clinch, Michael J Newton, Stephen B Dunnett, Sean L Wyatt, Anwen S Williams, Simon A Jones

**Affiliations:** 1 Division of Infection & Immunity, School of Medicine, Cardiff University, Cardiff CF14 4XN, UK; 2 School of Biosciences, Cardiff University, Museum Avenue, Cardiff, CF10 3AX, UK; 3 Arthritis Research UK Biomechanics and Bioengineering Centre, Cardiff University, UK

**Keywords:** Behavior, Arthritis, Inflammation, IL-10, Cytokine

## Abstract

**Introduction:**

Although rheumatoid arthritis (RA) is a disease of articular joints, patients often suffer from co-morbid neuropsychiatric changes, such as anxiety, that may reflect links between heightened systemic inflammation and abnormal regulation of the hypothalamic-pituitary-adrenal (HPA) axis. Here, we apply behavioral neuroscience methods to assess the impact of antigen-induced arthritis (AIA) on behavioral performance in wild type (WT) and interleukin-10 deficient (*Il10*^-/-^) mice. Our aim was to identify limb-specific motor impairments, as well as neuropsychological responses to inflammatory arthritis.

**Methods:**

Behavioral testing was performed longitudinally in WT and *Il10*^-/-^ mice before and after the induction of arthritic joint pathology. Footprint analysis, beam walking and open field assessment determined a range of motor, exploratory and anxiety-related parameters. Specific gene changes in HPA axis tissues were analyzed using qPCR.

**Results:**

Behavioral assessment revealed transient motor and exploratory impairments in mice receiving AIA, coinciding with joint swelling. Hind limb coordination deficits were independent of joint pathology. Behavioral impairments returned to baseline by 10 days post-AIA in WT mice. *Il10*^-/-^ mice demonstrated comparable levels of swelling and joint pathology as WT mice up to 15 days post-AIA, but systemic differences were evident in mRNA expression in HPA axis tissues from *Il10*^-/-^ mice post-AIA. Interestingly, the behavioral profile of *Il10*^-/-^ mice revealed a significantly longer time post-AIA for activity and anxiety-related behaviors to recover.

**Conclusions:**

The novel application of sensitive behavioral tasks has enabled dissociation between behaviors that occur due to transient joint-specific pathology and those generated by more subtle systemic alterations that manifest post-AIA.

## Introduction

Rheumatoid arthritis (RA) is an immune-mediated inflammatory disorder that manifests as painful articular disease and associated loss of joint function. Underlying the pathogenesis of RA is an imbalance in the complex network of pro- and anti-inflammatory cytokines, resulting in degenerative destruction of bone and articular cartilage. Whilst the pathophysiological symptoms of RA are cardinal signs of disease, there is evidence to suggest that a substantial burden of RA disease is linked to extra-articular comorbidities and psychosocial factors that may be mediated by systemic inflammatory processes [1].

To identify the extra-articular components of RA, it is necessary to validate novel methods capable of objectively assessing behavioral characteristics in inflammatory arthritis. Models of experimental arthritis with well-defined joint pathology [2–4], in combination with behavioral assessment, would enable classification of behaviors as dependent or independent of disease severity. Behavioral assessment within experimental arthritis research has, to date, been limited and focused primarily on gait evaluation [5–12]. Although open field test (OFT) techniques have been applied to experimental arthritis, these studies were limited by measuring activity at a single time-point [13,14] rather than in response to arthritis induction or progression. We propose that longitudinal assessment, using complementary tests, would deliver a more complete picture of behavior during experimental arthritis. Such an approach would reveal global behavioral attributes driven by more subtle systemic changes, which may manifest independently from joint-specific processes.

Important considerations in assessing behavior in rodent models of RA are the classification and severity of disease. Poly-arthritic models inherently affect the general state of health of the animal, rendering behavioral studies difficult to interpret. Mono-arthritic models, such as the antigen-induced arthritis (AIA) model [3], provide a more suitable framework for disentangling the relationship between disease severity and global behavior status in arthritis. Disease severity can be manipulated by disrupting cytokine profiles. For example, mice deficient in pro-inflammatory [15,16] and antiinflammatory [4,17,18] cytokines present with differing degrees of synovial histopathology. Ultimately, these findings have led to the clinical introduction of biological drugs that target specific cytokine activities [19,20].

In addition to their important roles in mediating inflammatory processes, cytokines have been linked to mood disorders such as anxiety and depression [21–28] and implicated in neuroendocrine regulation of the hypothalamus-pituitary-adrenal (HPA) axis [29,30]. For example, relative levels of HPA hormones have been shown to predict response to biologic therapy in RA [30]. Secondly, mice deficient in anti-inflammatory interleukin-10 (*Il10*) exhibit depressive-like behavior [24]. Thirdly, the pro-inflammatory cytokine interleukin-6 (*Il6*) is able to stimulate the HPA axis [21], and can act at the level of the adrenal cortex [22]. Finally, high levels of pro-inflammatory cytokines have been measured in patients with depression [31,23] and neuropsychiatric changes, including anxiety, fatigue and depression, are prevalent in RA [32,33]. In summary, there is abundant evidence for bidirectional neuroendocrine-inflammation crosstalk in RA. However, the relationship between disease severity, behavior/mood and cytokine action remains unclear.

The aim of our study was to validate a novel longitudinal approach for behavioral evaluation of experimental arthritis in rodents, both during the acute phase of AIA and during recovery from the inflammatory processes, but prior to the onset of overt joint disease. By employing a range of sensitive behavioral tasks, we sought to identify distinct profiles of behavior that manifest either after the acute, transient joint swelling or as a result of more subtle systemic changes that manifest post-AIA. To achieve this, we first compared wild-type (WT) mice with and without AIA to identify behavioral tasks sensitive to AIA. Secondly, we compared WT to *Il10*^-/-^ mice, which exhibited similar levels of swelling and joint pathology up to 15 days post-AIA as WT mice, but importantly, are thought to have different intrinsic immune responses [4]. Our decision to examine behavior up to, but not beyond, 15 days was based on our finding that joint pathology is comparable between WT and *Il10*^-/-^ mice up to 15 days post-AIA [4]. However, after that, changes in behavior may be driven by overt joint degeneration and not by the more subtle, systemic immune or hormonal alterations that manifest after AIA. Lastly, we used qPCR analysis of HPA axis tissues to reveal systemic changes that may underlie the extra-articular behavioral changes evident long-term in *Il10*^-/-^ mice post-AIA.

## Methods

### Mice

For the behavioral experiment, fourteen male WT C57BL/6 (Charles River, UK) and eight *Il10*^-/-^ (C57BL/6 background, Jackson Laboratory, ME) were housed in groups of 3-5 and maintained on a 14:10-h light/dark cycle, with food and water available *ad libitum*. Experiments were performed on 8–12 week old mice.

A second cohort of male mice, consisting of 6 WT and 6 *Il-10*^-/-^ mice, 8-12 weeks old, were utilised for the investigation of gene expression within the HPA axis 3 days post AIA injection. These mice were housed in identical conditions to those utilised for behavioral analysis.

All experiments using animals were conducted in compliance with the UK Animals (Scientific Procedures) Act 1986 and ethical approval was assured through approval of UK Home Office Project License PPL-30/2928.

### Antigen induced arthritis (AIA)

Mice were immunized by subcutaneous administration of 100 μl methylated bovine serum albumin (mBSA; 1 mg/ml, Sigma-Aldrich) emulsified in complete Freund’s adjuvant (CFA, Sigma-Aldrich). Mice were injected i.p. with 200 ng of heat-inactivated *Bordetella pertussis* toxin (Sigma-Aldrich). The immune response was boosted after 7 days with identical administration of mBSA/CFA. Inflammatory arthritis was induced 21 days after the initial immunizations by intra-articular administration of 10 μl mBSA (10 mg/ml) into the right hind knee joint. For the WT-PBS group, 10 μl phosphate buffered saline (PBS) was injected into the knee joint. Swelling was monitored by measuring knee joint diameter using a POCO 2T micrometer (Kroeplin), and recorded as the difference between right (inflamed) and left (contralateral) diameters. Knee joints were harvested at day 15 post-AIA for histopathological assessment of disease severity (arthritic index).

### Longitudinal behavioral assessment

A within-subjects design was chosen for this study, in order to observe the effect of arthritis onset within the same mouse. A withinsubjects design is particularly robust insofar as a direct comparison can be made with and without arthritis within the same host. WT mice were randomly assigned to receive AIA or PBS. All *Il10*^-/-^ mice received AIA.

**Figure 1:**
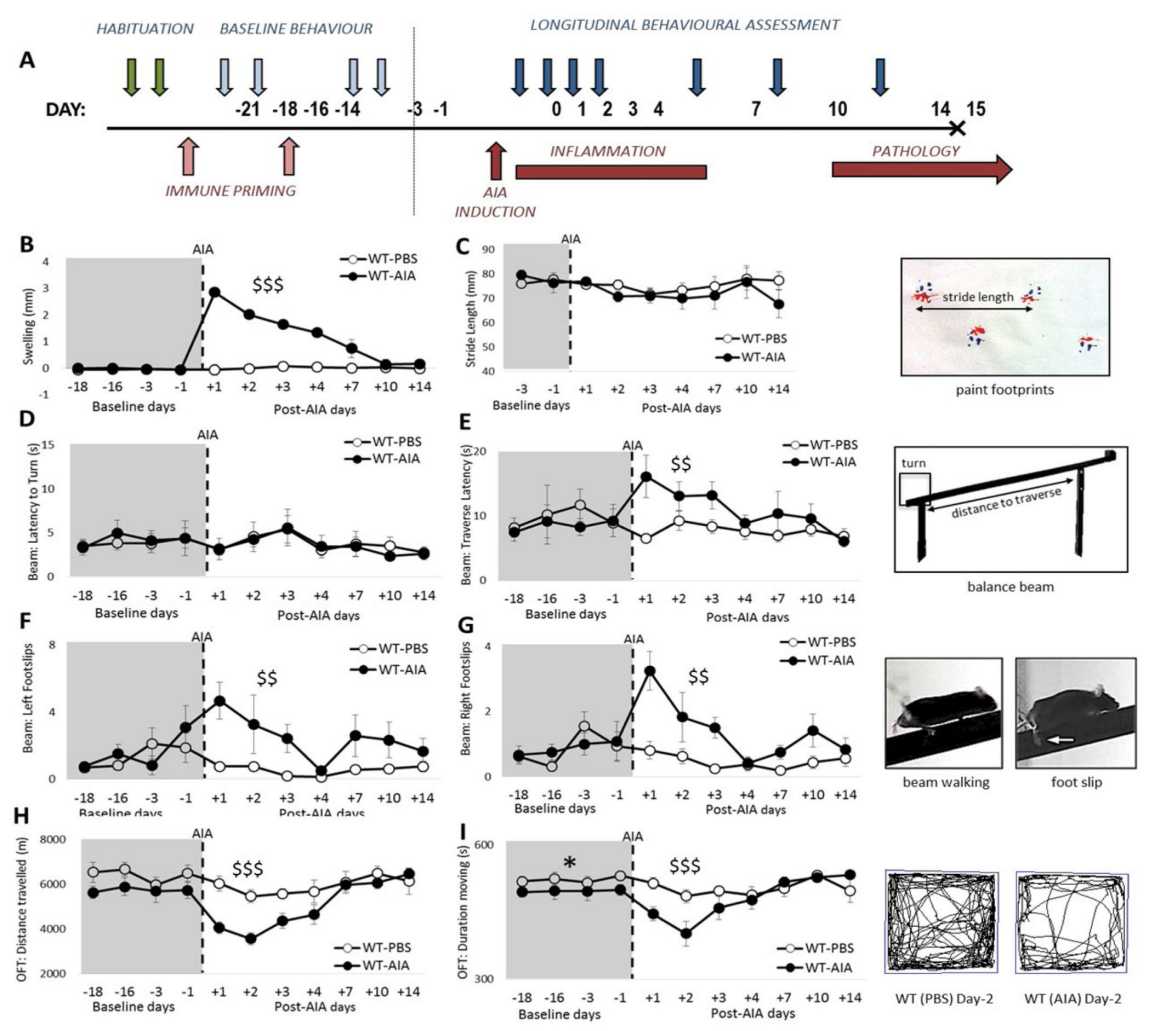
Novel application of combined behavioral measures to assess motor function and locomotor activity in experimental arthritis. (**A**) Schematic representation of longitudinal behavioral assessment during AIA. Blue arrows denote habituation to the behavioral tasks, 4 sessions of baseline measurement, and 7 testing days; (**B**) Significantly greater knee swelling in AIA-injected mice compared to PBS-injected mice; (**C**) No significant effect of PBS or AIA injection on stride length; (**D**) Latency to turn is not significantly different between the groups; (**E**) AIA-treated mice transiently demonstrate significantly longer latency to traverse the balance beam than PBS-treated mice; (**F&G**) AIA injection induced a significant transient increase in the number of foot slips made by both hind limbs; (**H**) Open field testing revealed a transient reduction in total distance moved and (**I**) time spent moving in the AIA-treated group compared to the PBS-treated group. Tracks show representative movement (black line) on day 2 post injection, when the AIA-injected group are significantly less active that the PBS-injected group. B–baseline testing day; Graphs represent mean ± SEM; Significant effect of Group: *p ≤ 0.05; Significant Group*Day interaction: ^$$^p ≤ 0.01, ^$$$^p ≤ 0.001.

All mice were habituated to the open field apparatus over two days prior to the start of behavioral testing (Figure 1A). To establish the baseline behaviors of WT and *Il10*^-/-^ mice in open field tests (OFT) and balance beam assessments, all mice were tested 4 times, over a period of 18 days, prior to the onset of AIA. Two behavioral tests were conducted before the immunization with BSA, and two behavioral tests were conducted after the mBSA injections (Figure 1A). For footprint analysis, two sessions were conducted to obtain baseline data, at 3 days and 1 day pre-AIA induction. Behavior was found to be consistent over this 18 day pre-AIA period for both genotypes on all tasks (see results section below for statistical analyses).

Each day, mice were assessed in the same order: animal distress rating/weighing, balance beam, OFT, footprint analysis. Four successive time-points post-AIA were chosen to capture behavioral alterations associated with acute inflammation, followed by 3 further time points up to 14 days post-AIA. Thus, behavioral testing was performed over 4 sessions during the 18 day pre-AIA period and for 7 sessions over the 14 day post-AIA period (Figure 1A). Mice were perfused on day 15. The end-point, day 15, was determined by previously published data showing that up to day 15 post-AIA, *Il10*^-/-^ mice were indistinguishable from WT mice on all measures of the arthritic index [4]. However, given that Greenhill et al. report that long-term differences in the extent of disease pathology emerge by day 28 post-AIA [4], we hypothesized that subtle, perhaps systemic, changes induced in the *Il10*^-/-^ mice by the AIA may be revealed using the behavioral assays utilized here, which haven’t previously been detectable.

### Elevated balance beam

The apparatus consisted of a tapered beam (0.5-1.5 cm) with a 0.5 cm ledge that was situated 2 cm below the running surface. The bean was 100 cm long and angled at 17°. Mice were pre-trained to run up the beam to a goal-box. During testing, mice were placed at the lower end, facing away from the running surface. The turning time was recorded as a measure of motor coordination and the time to traverse indexed walking difficulty. The number of foot slips made by each limb was recorded as a measure of balance. Each mouse underwent three trials per day.

### Open field test

Mice were placed in the middle of the arena (80 × 80 × 40 cm) and allowed to explore for 10 min. The arena was uniformly illuminated and the arena was always located in the same room. Two “locomotor” parameters were calculated: distance travelled and duration moving. After dividing the OFT arena into a central and a peripheral zone, two further anxiety-related parameters were calculated: the amount of time spent within, and the number of entries into, the central zone.

### Monitoring of behavioral responses in the open field test

Behavior was recorded by a video camera, attached to a PC-based data recorder, and analyzed using EthoVision tracking software (Noldus Information Systems, The Netherlands).

### Footprint analysis

Mice walked along a custom-built walkway on strips of paper (60 × 10 cm). Their front and hind paws were painted with red and blue paint, respectively. Three series were recorded per day. Stride length was defined as the distance between feet of the left and right stepping cycle.

### Animal Distress Score

An animal Distress Scoring system was used to monitor changes in health and wellbeing pre- and post-AIA in WT and *Il10*^-/-^ mice [34]. This rating system considers several aspects of health, including weight, appearance, clinical symptoms, natural behavior and provoked behavior. Each category has a scale of 0-3, with ‘0’ denoting normal health and ‘3’ conveying a severe health condition. The appearance of the mouse may score ‘0’ if normal, ‘1’ if a general lack of grooming, ‘2’ where coat starring and/or ocular or nasal discharge is evident. ‘3’ when piloerection and hunching are observed. Clinical symptoms may not be present (0), or may be slight (1; pallor, diminished activity), moderate (2; weight loss, polyurea, diarrhoea) or severe (3; immobility, abnormal gait, fitting). Assessment of natural behaviors may reveal (0) normal behavior, (1) minor changes, (2) less mobile/alert, isolated or (3) vocalization, self-mutilation, restlessness/stillness. Lastly, Provoked behavior may be (0) normal, (1) slightly depressed or exaggerated response, (2) moderate changes in expected behavior or (3) a violent reaction or very weak/precomatose response. Weights are also monitored and rated if changed. Each day, the score is adjusted if a 3 is assigned more than once, by adding 1 point per 3 score. Finally, 0-4 is considered normal behavior, 5-9 reveals that the mouse requires carefully daily monitoring and > 10 determines suffering and the need for termination.

### Histology

Joints were fixed in neutral buffered formalin saline, decalcified with formic acid at 4°C and embedded in paraffin. Parasagittal sections (8 μm) were stained with hematoxylin, safranin-O and fast green. Disease severity was scored according to arthritic index, which comprises four components: sub-synovial inflammation (0‑5); synovial exudate (0‑3); hyperplasia and pannus formation (0‑3) and articular cartilage or sub-chondral bone erosion (0‑3). Scores were taken as the average of two observers blinded to the experimental design.

### HPA axis evaluation by Quantitative RT-PCR

A second cohort of WT and *Il-10*^-/-^ mice were used to investigate the impact of IL-10 deficiency on gene expression within the HPA axis following AIA. 6 mice of each genotype received AIA injections. Mice were euthanized by cervical dislocation 3 days post-AIA and the hypothalamus, pituitary and adrenal cortex were rapidly dissected and placed into RNA*later* (Ambion) to inactivate RNAses. Following an overnight incubation at 4°C, tissues in RNA*later* were transferred to a -80°C freezer for storage until RNA extraction. Total RNA was extracted from dissected tissues using the RNeasy lipid extraction kit (Qiagen, UK).

### Quantitative PCR

200 ng of total RNA was reverse transcribed in a 20 ul reaction for 1h at 45°C using the AffinityScript kit (Agilent, UK). 2 μl cDNA was amplified in a 20 μl reaction volume using Brilliant III ultrafast qPCR master mix reagents (Agilent). PCR products were detected using dual-labeled (FAM/BHQ1) hybridization probes specific to each of the cDNAs (MWG/Eurofins, Germany). Gene changes in neuroendocrine signatures within tissues from the HPA axis were assessed by qPCR at day 3 following AIA onset. Expression of *Nr3c1*, *Crh*, *Tnfrsf1a*, *Pomc*, *Mc2r* and *Il6* was quantified in HPA axis RNA samples relative to a geometric mean of mRNAs for the reference genes *Gapdh*, *Sdha* and *Hprt1*. See Supplementary 1 for oligonucleotide primer sequences and standards. Oligonucleotide primers were used at a concentration of 200 nM. Dual-labeled probes were used at 500 nM. PCR was performed using the M × 3000P platform (Agilent) using the following conditions: 45 cycles of 95°C for 12s and 60°C for 35s.

### Statistics

Statistical analyses were performed using Prism (v6, GraphPad Software Inc.) and SPSS (v20, IBM). Behavioral and swelling data were analyzed using Repeated Measures ANOVA, with Group and Day as factors. Where an interaction between Group × Day was found to be significant, analysis of the simple effects was undertaken with a restricted ANOVA. Post-hoc effects were analyzed with the addition of a bonferroni correction for multiple comparisons, to ensure appropriate stringency. Histological data were analyzed using Univariate ANOVA, with Group as the between-subjects factor. The qPCR data were analyzed using unpaired t-test. Swelling and behavioral data are graphically depicted with the full 18 day pre-AIA period (consisting of 4 baseline behavioral test sessions) to demonstrate the stability of the behavioral performance of the WT and *Il10*^-/-^ mice. The 14 day post-AIA period (consisting of 7 test days) is then depicted. An analysis was conducted on the baseline data to determine the pattern of performance on the task prior to AIA onset and to identify any baseline differences between WT and *Il10*^-/-^ mice. Then an analysis of the post-AIA data was conducted to determine the effect of the AIA insult on behavioral performance.

**Figure 2:**
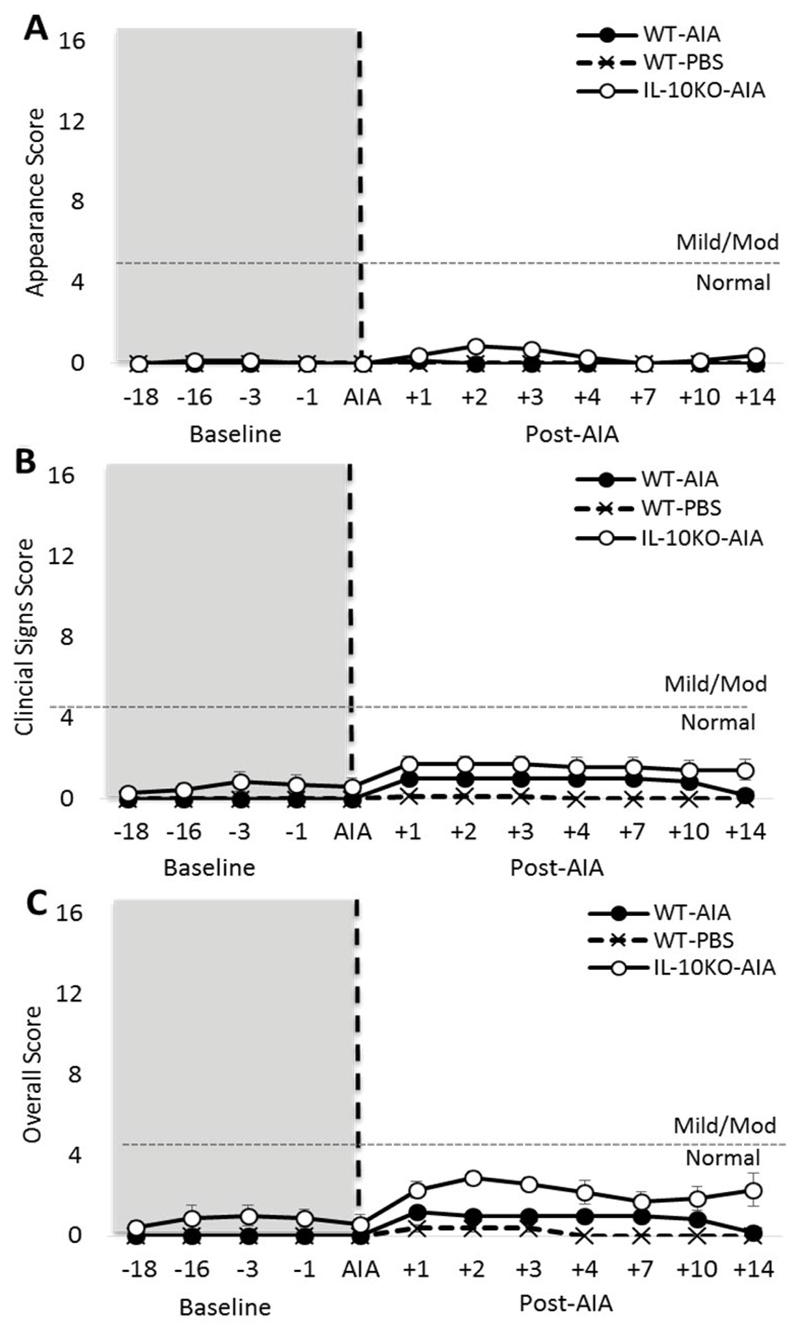
Induction of AIA generates subtle clinical symptoms in IL-10^-/-^ mice. (**A**) Application of the Animal Distress Scoring system revealed no difference in appearance at baseline, but a subtle change in appearance in IL-10^-/-^ mice after AIA; (**B**) Very mild clinical symptoms were observed in IL-10^-/-^ mice at baseline, but these fell within the range of normal behaviors (0-4 score). Post-AIA, both WT and IL-10^-/-^ mice presented with a mild increase in clinical symptoms, which were sufficiently subtle to remain within the range of normal behaviors. No changes were observed post-AIA for either genotype on the categories of weight, natural behaviors or provoked behaviors (data not shown). Significant effect of Group: *p ≤ 0.05; Significant Group*Day interaction: ^$$$^p ≤ 0.001.

To investigate the interaction between loss of IL-10 and arthritis induction on behavioral performance, an analysis compared data obtained during the baseline training against data obtained from the final week of AIA (Figure 2). Datasets from all four days within the baseline period (-18, -16, -3, -1) were averaged and compared against the average of datasets for the final two days of assessment post-AIA (+10, +14). The percentage change post-AIA, relative to the baseline period, was calculated. Independent samples t-tests explored differences in performance between the genotypes.

## Results

### Identification of acute behavioral impairments after AIA induction in WT mice

Schematic depicting the experimental design for longitudinal assessment of AIA (Figure 1). At baseline (prior to AIA onset), no differences in swelling, stride length, performance on the balance beam, nor distance travelled in the OFT were evident between the WT groups [maximum: F_1,12_ = 2.81, n.s.]. Although a mild but significant difference was revealed on the duration of time spent moving in the OFT [Group: F_1,12_ = 6.08, p < 0.05], overall the groups were well matched at baseline.

Mice receiving i.a. injection of mBSA (WT-AIA) displayed significant joint swelling from day 1 post AIA. While acute alterations in joint swelling continued for 7 days post arthritis induction, this response showed no relationship to changes in behavior. In contrast, control mice receiving PBS (WT-PBS) showed no joint swelling [Figure 1B; Group: F_1,12_ = 69.12, p < 0.001; Group × Day: F_7,84_ = 45.58, p < 0.001].

While neither the stride length [Figure 1C; F < 1], nor the latency to turn [Figure 1D; F < 1] on the balance beam were affected by antigen challenge, WT mice with AIA were slower to traverse the length of the balance beam than WT-PBS mice, an effect that lasted until day 4 of AIA [Figure 1E; Group × Day: F_7,84_ = 6.72, p < 0.01]. WT-AIA mice demonstrated a significantly higher number of foot slips of both the ipsilateral (right) and the contralateral (left) hind limbs, an effect which lasted for 3 days post-AIA in both hind limbs [Figure 1(F&G); min: Group × Day: F_7,84_ = 3.30, p < 0.01]. This motor deficit was specific to the hind limbs. No significant increase in the number of foot slips were observed for the front limbs after antigen challenge (data not shown; maximum F_1,12_ = 2.49, p > 0.05). In OFT assessments, the total distance travelled was transiently reduced by the onset of AIA, returning to baseline levels by 4 days post-insult [Figure 1F; Group × Day: F_7,84_ = 4.38, p < 0.001] and the duration of time spent moving was shorter in WT-AIA mice [Group × Day: F_7,84_ = 4.27, p < 0.001]. The indices of anxiety (quantified as entries into the inner zone and time spent in the inner zone) were not significantly altered after AIA [data not shown; maximum: Group: F_1,12_ = 2.70, all p’s n.s.].

All behavioral abnormalities in WT-AIA mice returned back to baseline by day 7 post AIA, when joint swelling had subsided.

### Similar motor coordination deficits and articular joint pathology during acute AIA in WT and IL-10-deficient mice

**Figure 3:**
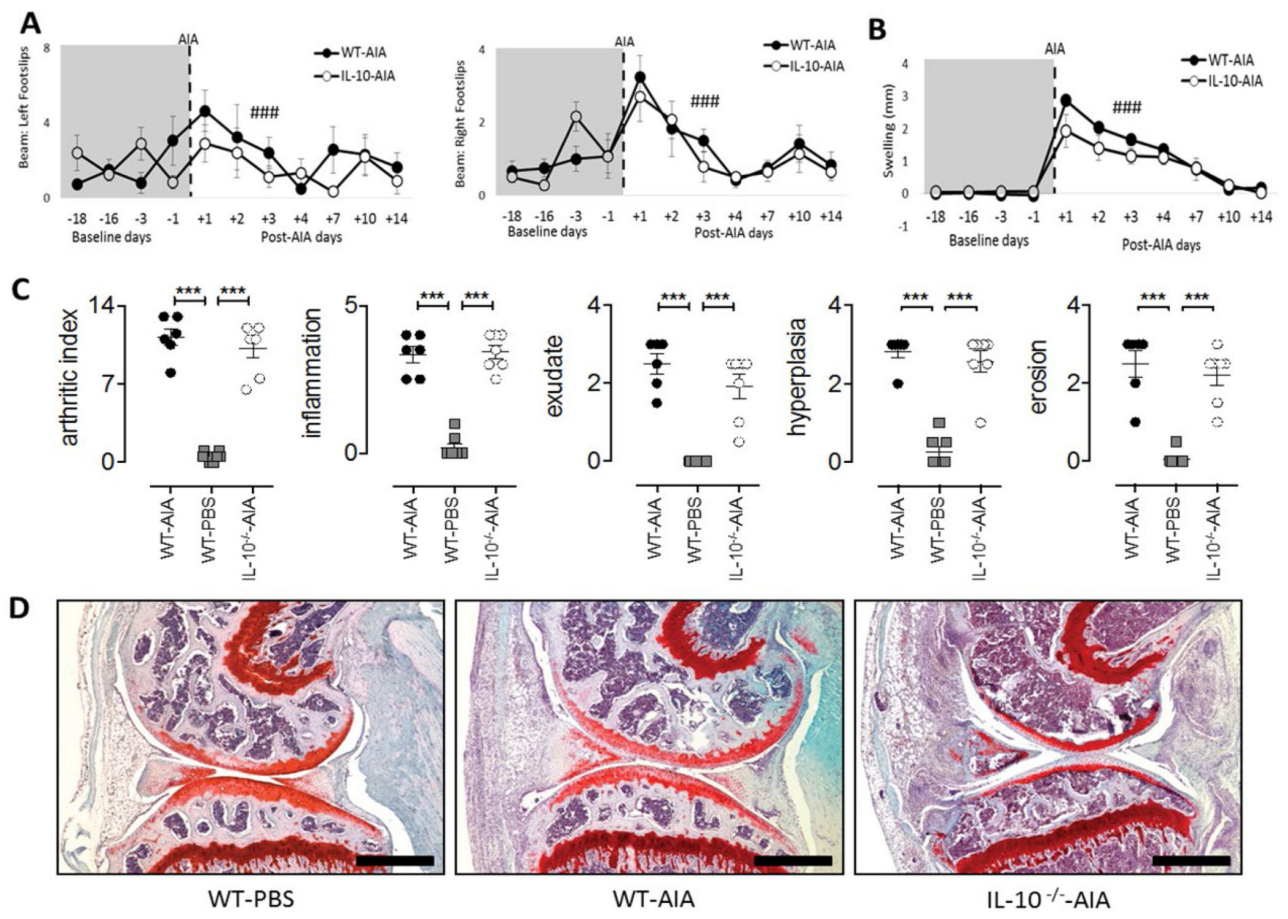
Joint-specific processes during acute AIA are independent of IL-10. (**A**) Significant transient increase in number of footslips post AIA in both WT and IL-10^-/-^ groups; (**B**) Significant increase in swelling due to AIA induction in both WT and IL-10^-/-^ groups; (**C**) Evaluation of arthritic index, inflammation, exudate, hyperplasia, and erosion in AIA-induced knee joints of WT and IL-10^-/-^ mice, compared to WT-PBS treated mice. Values are presented for individual joints taken at Day-15 post AIA; (**D**) Representative Safranin-O and Fast Green stained parasagittal joint sections taken on Day-15 for WT-PBS, WT-AIA and IL-10^-/-^-AIA mice (scale bars, 500 μM). B–baseline testing day. Graphs represent mean ± SEM. Significant effect of Day: ^###^p ≤ 0.001; significant effect of Group: ***p ≤ 0.001.

There were found no overt differences in joint swelling or articular pathology as assessed by measurement of inflammatory infiltration and exudate, synovial hyperplasia and joint erosion in WT and *Il10*^-/-^ mice. While the induction of disease in WT and *Il10*^-/-^ mice promoted changes in joint swelling [maximum: F_1,11_ = 3.36, n.s.] and synovial inflammation [Figure 3(C&D); minimum: Group: F_2,28_ = 35.33, all p’s < 0.001; WT and *Il10*^-/-^ AIA vs. WT-PBS, all p’s < 0.001], no difference in disease severity was observed between the two mouse genotypes [Figure 3(C&D); WTAIA vs *Il10*^-/-^ AIA, all p’s n.s.]. On the balance beam test, the number of foot slips coincided with the development of knee swelling in the AIA treated right hind joint. Both *Il10*^-/-^ and WT mice showed a similar number of foot slips in response to AIA [maximum: Group: F_1,11_ = 3.36, n.s.]. All mice slipped more often on the balance beam with both hind limbs [Figure 3A; minimum: Day: F_6,66_ = 8.75, p < 0.001] and significant swelling was observed in the knee joint after AIA induction [Figure 3B; Day: F_6.66_ = 85.45, p < 0.001].

### Arthritis onset has a minimal impact on animal welfare

Application of the Animal Distress Scoring system revealed no substantive difference between WT and *Il10*^-/-^ mice on either ‘natural’ or ‘provoked’ behaviors. This was true both before and after the onset of AIA [data not shown; maximum: Group: F_2,28_ = 3.25, n.s.]. A small difference was observed on the ‘clinical signs’ score between WT and *Il10*^-/-^ mice at baseline [Figure 2A; Group: F_2,18_ = 5.05, p < 0.05]. However, this was extremely subtle and equated to < 1 on the clinical symptoms scale (0-4 = normal, 5-10 = mild symptoms).

However, following AIA onset, both WT-AIA and *Il10*^-/-^ AIA mice experienced a fluctuation in clinical symptoms that reached approximately 1.7 on the scale, demonstrating a very mild effect of AIA on wellbeing [Group*Day: F_14,126_ = 3.39, p < 0.001]. No significant differences were revealed between WT-AIA and *Il10*^-/-^ AIA mice, but both these groups receiving AIA differed from WT-PBS mice.

The appearance rating did not differ between the groups at baseline [Figure 2B; Group: F_2,18_ = 1.00, n.s.]. However, post-AIA, there was a transient and subtle impact of the intervention on the appearance rating of *Il10*^-/-^ mice only [Group*Day: F_14,126_ = 3.16, p = 0.001].

Slight changes in appearance were also evident post-AIA in *Il10*^-/-^ AIA mice only. These changes accounted for the difference in overall score observed at baseline [Figure 2C; Group: F_2,18_ = 7.56, p < 0.01] and post-AIA [Group*Day: F_14,126_ = 2.87, p = 0.001].

### Arthritis induction promotes extra-articular changes in mobility and anxiety in *Il10*^-/-^ mice

#### Baseline behavioral performance

**Figure 4:**
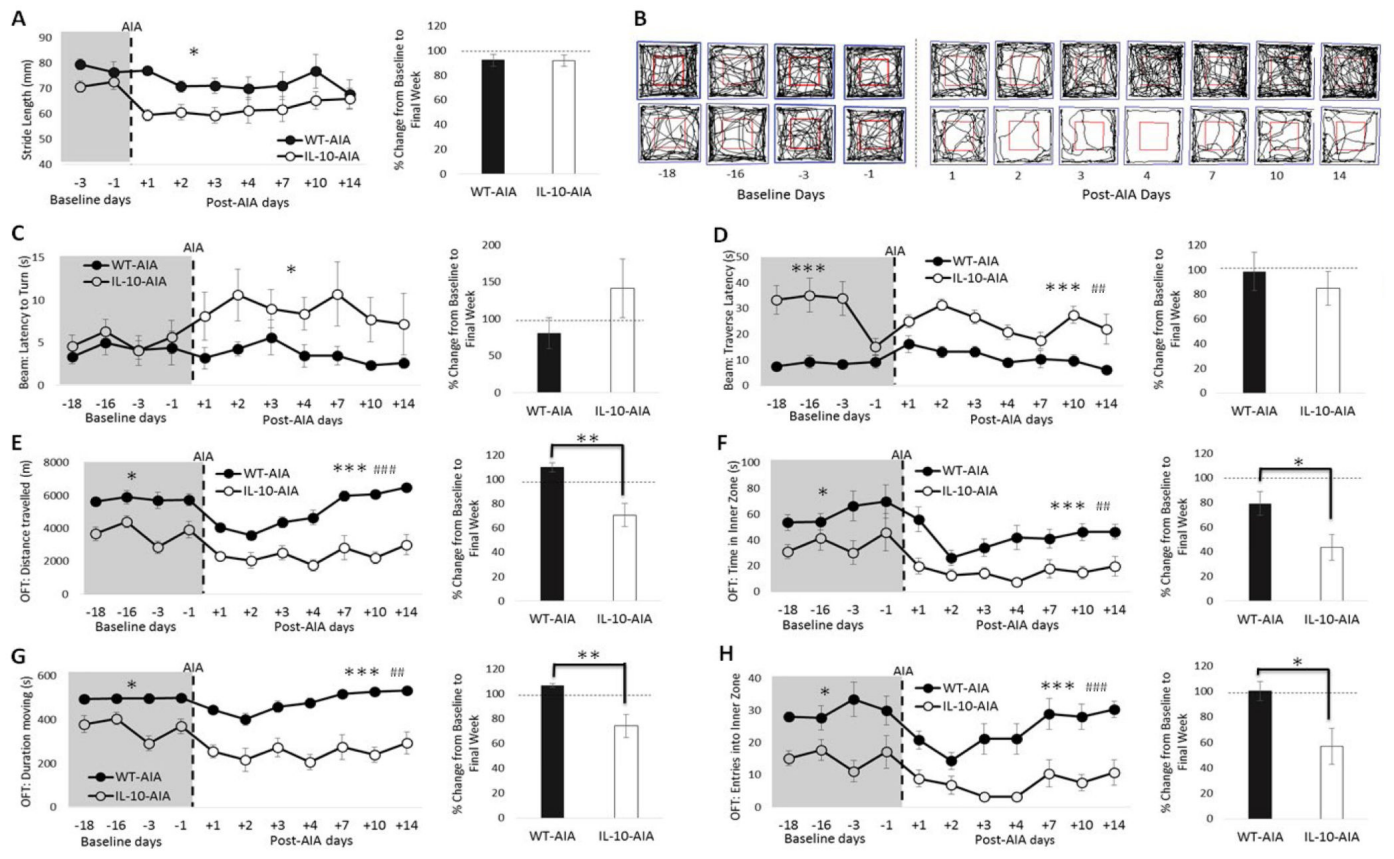
Loss of IL-10 delays recovery of global behavioral deficits induced by AIA. (**A**) Significant reduction in stride length by IL-10^-/-^ mice after AIA induction, which returns to baseline levels by the final week of testing; (**C**) The latency to turn on the balance beam is impaired post-AIA in IL-10^-/-^-AIA mice but not WT mice; (**D**) IL-10^-/-^-AIA mice take significantly longer than WT-AIA mice to traverse the balance beam at baseline and post-AIA; (**B**) Representative movement tracks (black line) of one WT-AIA and one IL-10^-/-^-AIA mouse in OFT. Blue square represents arena border and red square denotes the inner and outer zones used to determine anxiety indices. (**E&G**) In the OFT, IL-10^-/-^-AIA mice spent less time moving and travelled shorter distances both pre-AIA. However, locomotor behavior is disrupted in both WT and IL-10^-/-^ mice post-AIA. Moreover, IL-10^-/-^-AIA mice take longer to recover the activity deficit; (**F&H**) In the OFT, the indices of anxiety reveal overall differences between the genotypes, but both WT and IL-10^-/-^-AIA mice demonstrate reduced time in, and entries into, the inner zone of the arena after AIA. IL-10^-/-^-AIA mice show prolonged impairment on these measures. Graphs represent mean ± SEM. Significant effect of Group: *p ≤ 0.05, **p ≤ 0.01, ***p ≤ 0.001; Significant effect of Day: ^##^p ≤ 0.01, ^###^p ≤ 0.001.

Prior to arthritic insult (at baseline), the loss of IL-10 had no effect on stride length nor on the time to turn on the balance beam [Figure 4(A&C); maximum: Group: F_1,11_ = 6.07, n.s.]. The latency to traverse the balance beam was increased in *Il10*^-/-^ AIA mice relative to WT-AIA mice [Figure 4D; Group: F_1,11_ = 70.16, p < 0.001]. Moreover, in the OFT, Il-10-/--AIA mice spent less time moving, travelled less far, entered into the inner zone fewer times and spent less time in the inner zone than WT-AIA mice [Figure 4(B,E&F); minimum: Group: F_1,11_ = 4.87, p = 0.05].

Importantly, however, prior to AIA insult, the behavior of the *Il10*^-/-^ and WT mice was found to be consistent and stable across the 18 baseline days on every behavioral task [maximum, both for OFT duration moving: Day: F_3,33_ = 2.84, n.s.; Day*Group: F_3,33_ = 2.83, n.s.]. The reduction in the latency to traverse the balance beam observed during the fourth session, after the elevated scores obtained in the first 3 sessions, is likely accounted for by improved experimenter technique with repeated practice.

#### Post-AIA behavioral performance:

Stride length, as measured in the footprint test, was specifically reduced in *Il-10*^-/-^-AIA mice, relative to WT-AIA mice [Figure 4A; Group: F_1,11_ = 5.46, p < 0.05]. By the final week, however, performance had returned to baseline for both genotypes [t_11_ = -0.05, n.s.]. Stance base width and hindpaw/forepaw overlap were also measured throughout this experiment. No effects of IL-10 deficiency, nor effects of AIA, were evident on these aspects of gait (data not shown).

On the balance beam, the latency to turn was significantly increased in *Il-10*^-/-^ mice post-AIA, relative to WT-AIA mice [Figure 4C; Group: F_1,11_ = 5.02, p < 0.05]. Although by the final week of testing, a numerical increase in latency to turn was still evident in *Il10*^-/-^ AIA mice, this effect did not reach conventional levels of statistical significance [t_11_ = 1.28, n.s.].

*Il10*^-/-^ AIA mice demonstrated an increased latency to traverse the beam [Figure 4D; Group: F_1,11_ = 22.50, p = 0.001], which was also evident at baseline. More interestingly, the significant effect of Day reveals the impact of AIA insult on performance [Day: F_6,6 6_= 3.82, p < 0.01]. Comparison of the latency data during the final week, as compared to the baseline period, reveals that both genotypes returned to baseline levels [t_11_ = -0.66, n.s.].

On the OFT, differences in performance were evident between the genotypes post-AIA on all measures, as was observed at baseline [Figure 4(B,E&F); minimum: Group: F_1,11_ = 15.06, p < 0.01]. Unlike at baseline, however, there was also a significant effect of Day on every measure, demonstrating the impact of the AIA on performance [minimum: Day: F_6,66_ = 3.52 p < 0.01]. Most importantly, on both measures of locomotor activity (distance travelled and duration moving), comparison of the activity levels during the final week, as compared to the baseline period, revealed a return to baseline performance for WT-AIA mice, while *Il10*^-/-^ AIA mice were still significantly affected by the AIA intervention during the second week post-AIA [t_11_ = -3.57&-3.18, respectively, p’s<0.01].

Moreover, on both measures of anxiety (time in inner zone and entries into inner zone), *Il10*^-/-^ AIA mice were impaired for longer than WT-AIA mice. By the final week of testing, the time spent in the inner zone was severely reduced in *Il10*^-/-^ -AIA mice, compared to WT-AIA mice [t_11_ = -2.48, p < 0.05]. Also, while the number of entries into the inner zone had returned to baseline levels for WT-AIA mice, the *Il10*^-/-^ AIA mice were still significantly impaired by the second week of post-AIA behavioral testing [t_11_ = -2.48, p < 0.05]. Overall, the results demonstrate that AIA induction initiates global, extraarticular behavioral abnormalities in both WT and *Il10*^-/-^ mice, and that specific subtle interactions between IL-10 deficiency and AIA can be detected on behavioral performance.

#### Up-regulation of specific HPA axis signatures is associated with IL-10 dependent increased anxiety index

**Figure 5:**
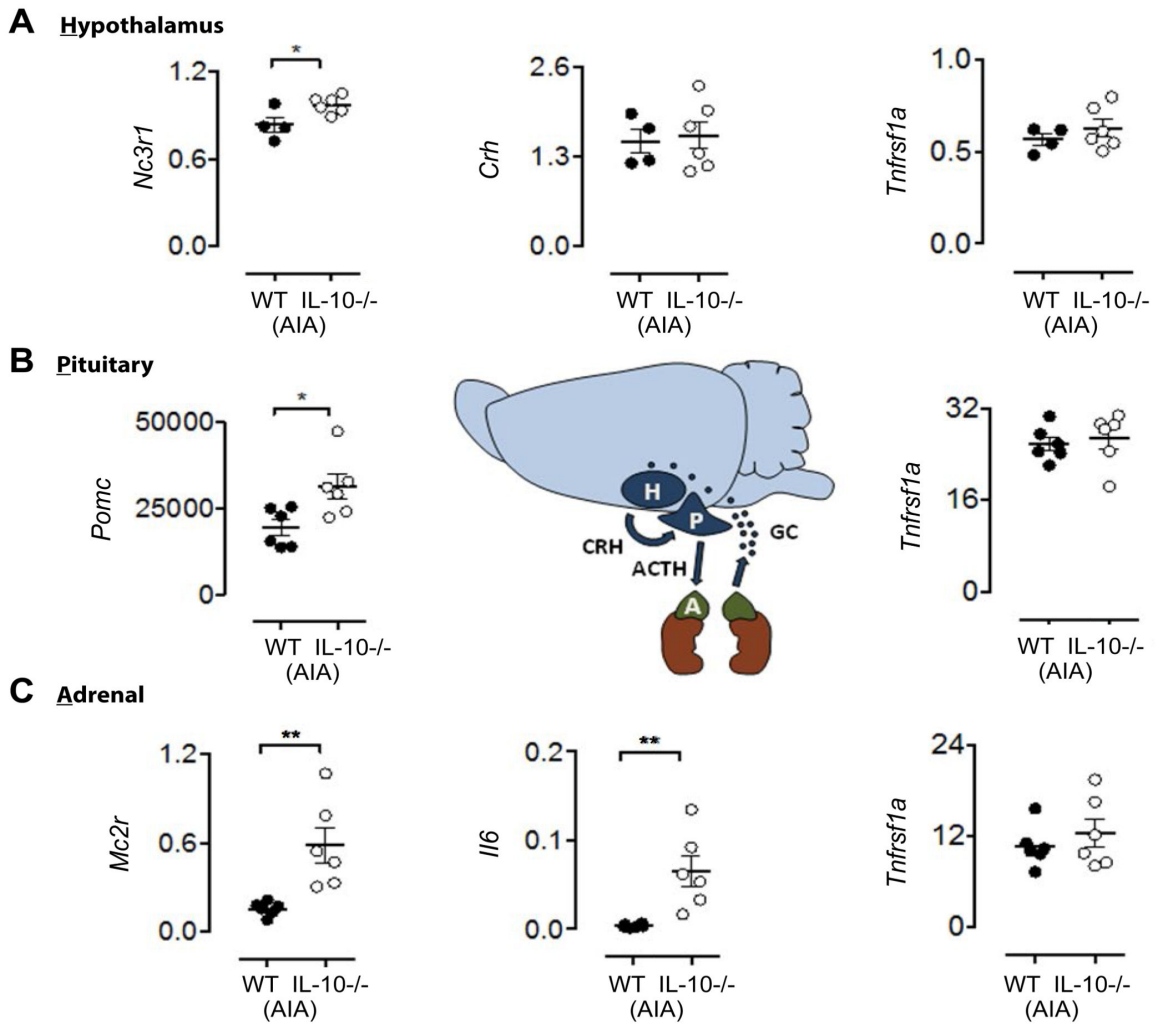
RT-qPCR of day HPA tissues 3 days post-AIA shows IL-10 dependent HPA axis mRNA signatures associated with increased levels of adrenal IL-6. (**A**) Significantly increased mRNA expression of hypothalamus glucocorticoid receptor (*Nc3r1*) in IL-10^-/-^-AIA mice. Expression of hypothalamic corticotropin-releasing hormone (*Crh*) and tumor necrosis factor receptor 1a (*Tnfrsf1a*) in IL-10^-/-^-AIA mice is not significantly different from WT-AIA mic; (**B**) Significantly increased mRNA expression of pituitary pro-opiomelanocortin (*Pomc*) in IL-10^-/-^-AIA mice. Expression of pituitary tumor necrosis factor receptor 1a (*Tnfrsf1a*) in IL-10^-/-^-AIA mice is not significantly different from WT-AIA mice; (**C**) Significantly increased mRNA expression of adrenal adrenocorticotropin receptor (*Mc2r*) in IL-10^-/-^-AIA mice and interleukin-6 (*Il6*). Expression of adrenal tumor necrosis factor receptor 1a (*Tnfrsf1a*) in IL-10^-/-^-AIA mice is not significantly different from WT-AIA mice. Graphs show all data points, with lines representing mean ± SEM. Significant effect of Group: *p ≤ 0.05, **p ≤ 0.01.

At day 3 post AIA induction, mRNA expression of specific HPA axis regulators was elevated in *Il10*^-/-^ -AIA mice compared to WT-AIA controls: hypothalamic glucocorticoid receptor, *Nc3r1* [Figure 5A, t_8_ = 2.63, p < 0.05]; pituitary pro-opiomelanocortin, *Pomc* [Figure 5B, t_10_ = 2.74, p < 0.05]; and adrenal cortex adrenocorticotropin hormone receptor, *Mc2r* [Figure 5C, t_10_ = 3.52, p < 0.01]. Other HPA signatures such as the precursor peptide for corticotropin-releasing hormone precursor, *Crh*, were not significantly different at day 3 [Figure 5A, n.s.]. Importantly, the differences observed at day 3 of AIA are not simply explained by IL-10 deficiency, as there were no significant differences between genotypes in the absence of AIA (n.s., for all effects of genotype at baseline; data not shown). Notably, a concurrent increase in adrenal pro-inflammatory IL-6 expression, *Il6*, was measured in the *Il10*^-/-^ AIA group (Figure 5C, t_10_ = 3.55, p < 0.01), compared to the WT-AIA group. Other inflammatory markers, such as tumor necrosis factor receptor 1A, *TNFR1a*, were unaltered by IL10 deficiency (Figure 5, maximum t_8_ = 0.96, n.s.).

## Discussion

This study provides novel insight into rodent behavior during experimental arthritis by applying behavioral neuroscience methodologies for longitudinal assessment of AIA. We utilized the OFT, footprint analysis and the balance beam test to assess the impact of arthritic insult, and its interaction with loss of IL-10, on behavior over 14 days. This novel application of select behavioral tasks has revealed several interesting phenomena. Firstly, these tasks identified changes in behavior that occurred as a result of arthritis but, more importantly, they can distinguish between joint-specific processes and more global, systemic changes in behavior. Mapping a timeline of behavior over two weeks also reveals transient dynamic changes that occur prior to the onset of chronic alterations.

From this study, we conclude, firstly, that the onset of arthritis causes acute, but transient, changes in swelling and motor performance, which affect balance and specific limb use in WT-AIA mice. Consistent with previous reports [2–4], AIA caused a characteristic antigen-dependent swelling of injected knee joints. Results demonstrate that specific behavioral deficits coincide with acute indices of AIA, such as joint swelling. Thus, the novel application of complementary behavioral tasks in mice with experimental arthritis reveals acute alterations in motor and locomotor responses.

Secondly, *Il10*^-/-^ -AIA mice demonstrated comparable changes in specific joint dysfunction to WT-AIA mice, as measured by the balance beam, knee swelling measurements and articular pathology indices. Thus, while deficits related to AIA were evident in joint swelling and footslips measures, loss of *Il10* did not exacerbate these joint-specific processes during AIA.

Thirdly, arthritis onset has a minimal impact on animal welfare. Natural and provoked behaviors were not affected by the onset of AIA, but subtle changes in clinical symptoms and appearance were observed in WT-AIA and *Il10*^-/-^ AIA mice. It is worth noting, however, that the magnitude of these changes was minor, since normal wellbeing registers between 0-4 on the rating scale, with mild/moderate changes ranging in between 5-9. Appearance changes averaged<1 and clinical signs reached a maximum of 1.7 on the rating scale. Therefore, an interesting but subtle interaction between AIA and IL-10 deficiency was revealed using the Animal Distress Scale

Next, these data reveal, interestingly, arthritis induction promotes extra-articular changes in mobility and anxiety in *Il10*^-/-^ mice. *Il10*^-/-^ -AIA mice presented with more systemic, global changes in behavior, and recovery of function was prolonged relative to WTAIA mice. Over a longer term, *Il10*^-/-^ AIA mice displayed shortened stride length, increased latency to turn on and traverse up the balance beam, a reduction in exploratory behavior, and fewer entries into the inner area of the OFT apparatus. Previous studies have shown that mice deficient in the anti-inflammatory cytokine IL-10 show a more sustained, long-term arthritic disease as compared to WT mice [4]. However, the extent of disease activity seen in WT and *Il10*^-/-^ mice remains comparable during the first two weeks following arthritis induction [4]. Thus, these chronic behavioral alterations cannot simply be accounted for by direct changes in limb function since joint-specific impairments, such as knee swelling and hind limb foot slips, returned to baseline in a similar manner in WT-AIA and *Il10*^-/-^ AIA mice. The observation that *Il10*^-/-^ mice have prolonged behavioral and anxiety-related impairments post-AIA may be important for addressing the high prevalence of mood disorders in patients with RA [33,34]. *Il10* deficiency is known to have profound immune outcomes that may influence the inflammatory control of neuropsychological behaviors. In addition to its role in modulating the severity of chronic experimental arthritis [4,17], IL-10 has been linked with anxiety and mood in both rodents and patients [22,24]. Although IL-10 plays a role in RA [19] and has previously been linked with mood disorders [24–26], there has been insufficient evidence for a specific role for IL-10 in regulating behavior during inflammatory arthritis. Here, we show that IL-10 deficiency altered the global motor and locomotor phenotype in mice that received AIA. Taken together, these results suggest reduced activity and heightened anxiety levels due to the interaction of IL-10 deficiency and AIA.

Finally, we found that IL-10 dependent increases in anxiety are associated with the up-regulation of specific HPA axis signatures. The IL-10 dependent delay in recovery of global locomotor and anxietyrelated behaviors identified using open field and balance beam techniques (Figure 2) occurred in the absence of any overt differences in articular pathology at day 15 post AIA. Whereas WT mice begin recovery earlier, the *Il10*^-/-^ mice display longer-term behavioral impairments. We therefore hypothesized that the IL-10-dependent global behavioral abnormalities observed during AIA were mediated by systemic processes, occurring outside the joint. Specifically, we focused on neuroendocrine signatures from HPA axis tissues. The results revealed increased mRNA expression of the specific HPA axis regulators *Nc3r1*, *Pomc*, *Mc2r* in *Il10*^-/-^ -AIA mice compared to WT-AIA controls at day 3 post AIA induction. Moreover, specific regulation of neuroendocrine pathways by IL-6, as determined here, is supported by previous studies showing stimulation of the HPA axis by IL-6 [20–21].

Thus, in this study, it has been possible for the first time to determine the role of IL-10 in behavioral control during experimental arthritis. The observation that IL-10 deficiency delays the recovery of global behaviors, independent of specific joint impairment, may be explained by aberrant inflammatory activation in *Il10*^-/-^ mice [4,17], or abnormal cytokine control of neuroendocrine processes [25]. Addressing these types of questions is becoming increasingly important for understanding the extra-articular comorbidities [1] and psychosocial factors [33,35] associated with RA.

When exploring behavioral responses in rodent models of chronic disease it is essential to consider the nature of the inflammation (i.e., localised vs. systemic) and the severity of disease (i.e., the histopathology). Systemic models of inflammatory disease (e.g. polyarticular models of arthritis) often have a more severe impact on animal health and welfare. For example, these models often necessitate the use of analgesia in the protocol. This makes behavioral studies more challenging and difficult to interpret. A monoarticular model of antigen-induced arthritis (AIA) was therefore adopted, where synovitis is established in one knee. This model shows 98-100% penetrance in C57/bl6 mice and the development of disease is well-characterized [10,13,16]. While studies in AIA have documented changes in joint pathology, gait analysis and loss of joint movement, the impact of AIA on animal behavior has never been tested. Previous behavioral assessments of experimental arthritis were limited to either single-technique gait evaluation [5–12] or single-trial OFT assessment [13–14].

Finally, we propose a potential mechanism underlying the delayed recovery of global behaviors observed in *Il10*^-/-^ mice. RT-qPCR expression profiling of HPA axis tissue 3 days post-AIA identified IL-10-dependent changes in gene expression in each of the three tissues. Specifically, *Nc3r1*, *Pomc*, and *Mc2r* mRNAs were expressed at higher levels in *Il10*^-/-^ mice after AIA compared to WT-AIA mice. Whilst adrenal IL-6 mRNA expression was up-regulated at the same time-point in *Il10*^-/-^ mice, *TNFR1a* mRNA expression in all HPA tissues did not differ between WT and *Il10*^-/-^ mice. These data raise the possibility that, in the absence of IL-10, AIA elevates levels of IL-6 within the adrenal cortex that may act, potentially locally, to modulate gene expression within the HPA axis, thereby altering axis function [22]. Further validation of this hypothesis is required in order to determine the precise mechanism of action.

## Conclusions

In summary, we have shown that the application of behavioral techniques to experimental arthritis can reveal novel and interesting phenomena that could fundamentally shift the landscape of RA treatment. Inclusion of longitudinal behavioral assessment in our experimental design enabled us to disentangle behaviors which are driven by articular insult, from those induced by systemic, extra-articular changes that are more purely induced by interaction between IL-10 deficiency and AIA. Although we have focused on IL-10 deficiency in the first instance, there is great potential for this type of work to unravel new mechanisms to inform new strategies for reducing the extra-articular disease burden [1].

## Supplementary Information

Supplementary Information

## Abbreviations

AIA: Antigen Induced Arthritis
HPA: Hypothalamic Pituitary Adrenal
mRNA: Messenger Ribonucleic Acid
IL-10: Interleukin-10
Il10-/-: Interleukin-10 Deficient
OFT: Open Field Test
qPCR: Quantitative Polymerase Chain Reaction
RA: Rheumatoid Arthritis
WT: Wild-Type
