# Supplementary material for: Novel Application of Behavioral Assays Allows Dissociation of Joint Pathology from Systemic Extra-Articular Alterations Induced by Inflammatory Arthritis
Source: J Rheum Dis Treat. Author manuscript; Available in PMC 2023 May 22. (PMC7614560; doi:10.23937/2469-5726/1510033)
Supplement: Supplementary Information [file EMS175614-supplement-Supplementary_Information.pdf]

Supplementary Information

Quantitative PCR

For quantifying *Il6* and *Tnfrsf1a* expression, standard curves were generated for every real time PCR run for *Il6*, *Tnfrsf1a* and reference gene cDNAs, by using serial threefold dilutions of reverse transcribed adult mouse spleen total RNA (Zyagen, San Diego, USA). For quantifying *Nr3c1*, *Crh*, *Pomc and Mc2r* expression, standard curves were generated for every real time PCR run for *Nr3c1*, *Crh*, *Pomc*, *Mc2r* and reference gene cDNAs, by using serial threefold dilutions of reverse transcribed adult mouse brain total RNA (Zyagen, San Diego, USA). Relative mRNA levels were quantified in four to six separate sets of each tissue dissected from each genotype. Primer and probe sequences were designed using Beacon Designer software (Premier Biosoft, Palo Alto, USA).

Primer /probe sequences	Accession number	QPCR product length/bp
Crh forward: 5′-ATC TCA CCT TCC ACC TTC- 3′<br>*Crh* reverse: 5′-CAA CAT TTC ATT TCC CGA TAA-3′<br>*Crh* probe: 5'-FAM-CTC CAT CAG TTT CCT GTT GCT GT-BHQ1-3'	NM_205769.2	113
Il6 forward: 5′-CAG AAA CCG CTA TGA AGT-3′<br>*Il6* reverse: 5′-CTT GTG AAG TAG GGA AGG-3′<br>*Il6* probe: 5′- FAM-TTG TCA CCA GCA TCA GTC C-BHQ1-3′	NM_031168.1	99
Mc2r forward: 5′-TGA AGC ATA TTA TCA ATT CGT AT-3′<br>*Mc2r* reverse: 5′-ACT ACA TCA GGA CAA TCG-3′<br>*Mc2r* probe: 5′-FAM-CAC ACC AAT GAC ACC GCA AGA-BHQ1-3′	NM_001271716.1	73
Nr3c1 forward: 5'-GGT TGG AGA TCA TAC AGA-3'<br>*Nr3c1* reverse: 5'-CTT TGT AAT TCA GTG GAG AT-3'<br>*Nr3c1*probe: 5'-FAM-CAA GTG GAA ACC TGC TAT GCT-BHQ1-3'	NM_006525658.1	140
Pomc forward: 5′-AAG AAC GCC ATC ATC AAG-3′<br>*Pomc* reverse: 5′-TCC TAA CAC AGG TAA CTC TA-3′<br>*Pomc* probe: 5′-FAM-TAG AGG TCA TCA GCT CGC C-BHQ1-3′	NM_001278581.1	129
Tnfrsf1a forward: 5′-TTC CCA GAA TTA CCT CAG-3′<br>*Tnfrsf1a* reverse: 5′-AAC TGG TTC TCC TTA CAG-3′<br>*Tnfrsf1a* probe: 5′- FAM-CAC CGT GTC CTT GTC AGC-BHQ1-3′	NM_011609.4	123
Gapdh forward: 5′-GAG AAA CCT GCC AAG TAT G-3′<br>*Gapdh* reverse: 5′-GGA GTT GCT GTT GAA GTC-3′<br>*Gapdh* probe: 5′-FAM-AGA CAA CCT GGT CCT CAG TGT-BHQ1-3	NM_001289726.1	122
Hprt1 forward: 5′-TTA AGC AGT ACA GCC CCA AAA TG-3′<br>*Hprt1* reverse: 5′-AAG TCT GGC CTG TAT CCA ACA C-3′<br>*Hrpt1* probe: 5′-FAM-TCG AGA GGT CCT TTT CAC CAG CAA G-BHQ1-3′	NM_013556.2	85
Sdha forward: 5′-GGA ACA CTC CAA AAA CAG-3′<br>*Sdha* reverse: 5′-CCA CAG CAT CAA ATT CAT-3′<br>*Sdha* probe: 5′-FAM-CCT GCG GCT TTC ACT TCT CT-BHQ1-3	NM_023281.1	127
